# Water, Sanitation, and Hygiene Practices and Their Association With Childhood Diarrhoea in Rural Households of Mayurbhanj District, Odisha, India

**DOI:** 10.7759/cureus.29888

**Published:** 2022-10-03

**Authors:** Madhulipika Giri, Manas Ranjan Behera, Deepanjali Behera, Biswajit Mishra, Damodar Jena

**Affiliations:** 1 Epidemiology and Public Health, Utkal University, Bhubaneswar, IND; 2 School of Public Health, Kalinga Institute of Industrial Technology (KIIT) University, Bhubaneswar, IND; 3 Quality Assurance, Kalinga Institute of Industrial Technology (KIIT) University, Bhubaneswar, IND; 4 School of Rural Management, Kalinga Institute of Industrial Technology (KIIT) University, Bhubaneswar, IND

**Keywords:** odisha, hygiene practice, sanitation, childhood infections, childhood diarrhoea, rural households, wash practices

## Abstract

Introduction: Diarrhoeal disease is one of the major causes of childhood morbidity and mortality in India. It affects all ages, yet very few studies have been found regarding the young and older children affected by diarrhoea and its etiology. About 2.2 million deaths are attributed to diarrhoea alone in India every year. However, a large number of diarrhoeal cases may be avoided with proper sanitation and hygiene practices. The primary aim of this study was to assess the current water, sanitation, and hygiene (WASH) practises among mothers and diarrhoea among their children (6 months to 11 years) in rural households of the Mayurbhanj district, Odisha. Further, the association between WASH practises and childhood diarrhoeal disease was assessed.

Methods: A community-based cross-sectional survey was conducted among 430 mothers by using a pre-tested structured questionnaire adapted from previous studies. Data collection was done using the software Epicollect5 by the trained data collector. The data were further transformed to Statistical Package for Social Sciences (SPSS, IBM Corp., Armonk, NY) version 23 for analysis to find out potential risk factors. A multistage sampling technique was used to find subjects.

Results: Out of 430 households, 21.6% of respondents reported that their children were suffering from diarrhoea in the last two weeks from the day of the survey. Nearly 63.8% of respondents mentioned that they used to supply water as a principal source for household purposes. This study found that children of households near waste piles are more at risk of having diarrhoea than those households living far from the waste pile [AOR: 4.996; 95% CI: (2.173, 11.487)]. In the management of household wastes, households who are throwing waste here and there are 3.276 times more at risk of having diarrhoea than households who are managing the wastes by themselves by burning them outside the home [AOR: 3.276; 95%CI: (1.463, 7.042)]. In the disposal of child faeces, it was found that the household children’s faeces disposed of in the garbage or dumping site are 2.438 times more at risk of having diarrhoea than those who are flushing the faeces in the latrine [AOR: 2.438; 95%CI: (1.284, 4.631)]. Also, using footwear less often was found to be highly associated with an increase in the incidence of diarrhoea (AOR: 1.634; 95% CI (.815, 3.276).

Conclusion: Findings indicate that creating awareness about the benefits of proper management of household waste and using footwear on a regular basis is the priority to avoid childhood diarrhoea. Further, area-specific planning and programme allocation of resources is necessary to prevent childhood diarrhoea.

## Introduction

Diarrhoeal illness is a major public health issue all over the world [1], including in developing countries such as India. Approximately 2.2 million deaths are attributed to diarrhoea alone in India, out of which diarrhoeal deaths under the age of five are estimated to be around 1.87 million [2]. It is also the most common cause of childhood malnutrition [3]. One of the major causes of diarrhoea among children is inadequate access to water and sanitation [2]. Therefore, every child has the right to grow up in a clean and safe environment, including the right to safe drinking water, toilets, and basic sanitary practices. This gives the children a healthier life and allows them to thrive at their best. Also, significant amounts of diarrhoea can be avoided with proper sanitation and hygiene practises [2]. Many poor and rural regions of developing countries such as India, Nepal, Pakistan, South Africa, Tanzania, etc., continue to fail to prosper due to inadequate access to water and sanitation [4-5]. In addition, between 2005 and 2015 [6], although the number of child deaths due to diarrhoea decreased by one-third, deaths due to diarrhoea remained high in some of the poorest countries [6].

Diarrheal illness is a substantial source of morbidity in children aged 1 to 11 years [7]. It leads to the loss of approximately 72.8 million disability-adjusted life years (DALYs). It deprives households' economic conditions and increases the healthcare system's burden [8]. Despite this, nearly 525,000 children under the age of five die each year as a result of diarrhoea worldwide [3]. This estimate exceeds the total mortality from malaria, TB, and HIV/AIDS [9]. Several research studies on water, sanitation, and hygiene (WASH) practises and school students have found that inadequate WASH practises increase school absenteeism and lower school performance [10-11]. Children under the age of 11 are more vulnerable to diarrheal illnesses [10]. This may be avoided by practising basic hygiene and providing clean water [7,11].

According to India’s National Family Health Survey-5 (NFHS-5), the proportion of households with an improved drinking water source is 95.9%, whereas it is only 80.1 percent in the Mayurbhanj district of Odisha [12]. There is also a significant difference between India (70.2%) and Mayurbhanj districts (54.4%) in the variable like households having improved sanitation facilities [12-13]. Investment in the WASH strategy can influence multiple development sectors. A study showed that improved WASH practises yield an average of four dollars in profit for every dollar invested [14]. Such a study assists various developmental agencies in establishing a relationship with WASH that contributes to the improvement of children who are in vulnerable situations [15].

Several studies have been carried out on diarrhoea due to poor water sanitation and hygiene practises among under-five children, but very few studies have been conducted taking older children up to 11 years of age [15-16] into consideration in the Mayurbhanj district of Odisha state, India. Therefore, the objectives of the study are: (i) to assess the current WASH practises among the mothers of children in the age group of 6 months to 11 years in rural households of Mayurbhanj district, Odisha; and (ii) to find the association between WASH practises and childhood diarrhoea up to 11 years old in rural households of Mayurbhanj district, Odisha.

## Materials and methods

Study design and description of the study area

This study was a cross-sectional community-based household survey, conducted from January 2, 2021, to May 29, 2021. The survey was conducted in the rural households of the Mayurbhanj district, which has a population of 2,519,738, in the Odisha state of India. The study was conducted in a randomly selected block, Muruda, in the Mayurbhanj district. According to census data from 2011 to 2022, the Mayurbhanj district comprised 586,253 households, out of which 7.4% of households belonged to urban and 92.6% to rural residents [17].

Study participants, inclusion and exclusion criteria

The study participants consisted of mothers of children aged 6 months to 11 years in selected households of the studied village. Women aged 15 to over 50 years old with children aged 6 to 11 years old who agreed to give consent were included. Women who were severely ill and refused to participate in this study were excluded.

Sample size calculation

A sample size estimation formula Zα2P(1−P)/d2 was used [18], taking an estimate of the prevalence (P) of diarrhoea 50%, 95% confidence level, and 5% confidence absolute precision. Considering the 10% non-response rate, the total sample size became 422 totaling to 430.

Sampling technique and procedures

A two-stage sampling technique was used. First, the block Muruda was selected randomly from the 31 blocks listed in the Mayurbhanj district. Out of a total of 115 villages under the Muruda block [17,19], every 28th village was chosen to conduct the survey using the systematic random sampling method. Then, a sampling frame was prepared for a house-to-house survey of households in the selected villages (villages 28th, 56th, 84th, and 112th) by identifying a mother of children aged 6 months to 11 years. There were a total of 870 households containing 925 mothers of children aged 6 months to 11 years. A proportion-to-size allocation was made to determine the sample size required from each selected village.

The sampling units were households with at least one mother of reproductive age and children aged 6 months to 11 years. In the second stage, systematic random sampling with a fixed interval of 2 was used to select sampling unit households. However, for the selection of the first household, the simple random lottery method was used. One mother/caretaker of children aged 6 months to 11 years was interviewed in each household. In the case of more than one eligible child, emphasis was given to the child having a recent episode of diarrhoea; otherwise, the selection was made using the lottery method.

Tool development

A structured questionnaire developed for data collection from a previous study by UNICEF was used to collect demographic, socioeconomic, and WASH variables [20]. A minor modification was made in the questionnaire according to the pretesting of the questionnaire conducted in the Rasgobindapur block of the Mayurbhanj district. About 62 respondents were interviewed in the pretest.

The questionnaire contains four sections. Section 1 contains four questions of sociodemographic information that include age, educational qualification, the total income of the family, and occupation. Section 2 contains two questions about household information that include the age of the child and the type of household in which the respondent's family resides. Section 3 contains 21 questions on WASH variables that include eight questions on water factors, nine questions on sanitation factors, and four questions on hygiene practices, respectively. One question was constructed in Section 4 to assess the health condition of the children: 'Has anyone in your household <11 years of age had unusual diarrheal symptoms (watery/bloody diarrhoea for a few days) in the past two weeks?'. They replied with ‘yes’ or 'no’ to the question. A value of ‘1’ was set for respondents who mentioned ‘yes’ and 0 for those who mentioned 'no’. Each data was checked manually in order to ensure completeness of data, clarity, and uniformity of data.

Data collection and data analysis

The data collection was done by two trained public health professionals using the software Epicollect5. The questionnaire was translated into the local language of Odia by the native language expert to conduct the interviews easily. IBM SPSS Statistics 23 was used for the analysis of the data. Descriptive statistics of sociodemographic data were calculated. Binary logistic regression was done to find the association between WASH practises and childhood diarrhoea. In this study, the occurrence of diarrhoea among 6-month to 11-year-old children within the last two weeks from the day of data collection was considered as the outcome (dependent) variable and WASH practises as the exposure (independent) variable.

Ethical issues

This study was approved by the Kalinga Institute of Medical Sciences (KIMS) ethical committee, KIIT University, Bhubaneswar, Odisha (No: 602/2021). A token of ascent was taken from the village key informant to conduct and cooperate during the study. Consent was taken from each respondent household who agreed to participate at the participant’s convenience with the assurance of confidentiality. Any participant in or during the study was free to refuse and refrain at any time. The study was performed solely for the purpose of the beneficence of the study participant. Precautions were taken not to harm the respondent.

Operational definition

The World Health Organization (WHO) definition of diarrhoea was used in this study. Acute diarrhoea was defined as the passage of three or more abnormally loose, watery stools in 24 hours and lasting not exceeding 14 days, and chronic diarrhoea was defined as lasting more than 14 days along with those symptoms [21]. The study used the World Bank definition of ‘improved water source’ which is described as households having access to piped water and a tube well within the premises and using the same water for drinking and household purposes [22]. Similarly, ‘improved sanitation’ was defined as the toilet, which is designed to keep a separation between humans and excreta hygienically [23]. A waste pile is a pile of non-containerized solid, non-flowing hazardous waste that is used for storage or treatment. In this study, people who practise hand washing with soap typically four times a day that is before food, after food, before cooking, and after using the toilet are said to be hand hygienic [24].

## Results

A total of 430 households participated in the study. Most of the mothers/caretakers were in the age group of 15 to 29 years. Around 60.2% of mothers were illiterate; 42.6% of households depended on farming as a main source of income. In this study, 37.7% of children were aged six months to two years, 35.1% were aged two years to five years, and the rest, 27.2%, were aged 5 years to 11 years. Most of the households were kutcha type, i.e., 56.5% (Table 1).

**Table 1:** Frequency and percentage of sociodemographic and household information of rural households in Mayurbhanj district, Odisha (n=430)

Personal and household information	Frequency (N)	Percentage (%)
Age of the mother/caretaker		
15 years to 29 years	260	60.5
30 years to 49 years	130	30.2
Above 50 years	40	9.3
Educational qualification of mother/caretaker		
Illiterate	259	60.2
Primary	116	27.0
Secondary	23	5.3
Higher secondary	32	7.4
Total income of the family in rupees		
1000–5000	179	41.6
5001–10,000	191	44.4
>10,000	60	14
Occupation		
Farmer	183	42.6
Driver	101	23.5
Labourer	105	24.4
Formal job	41	9.5
Age of the child		
6 months to 2 years	162	37.7
2 years to 5 years	151	35.1
5 years to 11 years	117	27.2
Household type the respondent family is residing		
Kutcha	243	56.5
Pucka	109	25.3
Kutcha-pucka	78	18.1

The study found that only 45.3% of households have four to seven days of water supply in a week. There were only 38.6% of houses that got supply water for two to three days per week. About 36% of households responded that the amount of water they were receiving was not satisfying their needs; 11.2% of households responded to the use of drinking water for cooking purposes. Only 15.6% of households have access to drinking water within their dwelling premises (Table 2).

**Table 2:** Frequency and percentage related to water factor (n=430)

Water factor	Frequency (n)	Percentage (%)
The principal source of drinking water		
Supply water	283	65.8
Tube well	147	34.2
Sufficiency of available drinking water		
Yes	360	83.7
No	70	16.3
Drinking water taste		
Good	121	28.1
Acceptable	309	71.9
Water source for cooking		
Domestic water	286	66.5
Drinking water	48	11.2
Both	96	22.3
Supply of drinking water per week		
Not connected	69	16
4–7 days per week.	195	45.3
2–3 days per week	166	38.6
Supply of drinking water per day		
Not connected	69	16
Less than 4 hours per day.	361	84
Satisfaction on amount of water received		
Yes	275	64
No	155	36
Distance to the principal source of drinking water		
Within dwelling	67	15.6
Outside dwelling but within the premises	194	45.1
Outside premises: less than 0.2–0.5 km	136	31.6
More than 0.2–0.5 km	33	7.7

The study found 21.9% of households have no toilet. According to the survey, 19.8 percent are still practising open defecation. About 64% have no bathroom in their household. A maximum (61.2%) of household people dispose of child faeces in the garbage and dumping sites. 75.6% of households said ‘yes’ to stagnant water near their home. 43% of households said they made their own dustbin and kept it inside the house (Table 3).

**Table 3:** Frequency and percentage related to sanitation (n=430)

Sanitation factor	Frequency (n)	Percentage (%)
Toilet facility in your household		
Attached	48	11.2
Detached	288	67
No toilet	94	21.9
Toilet type		
Pour latrine	204	47.4
Pit latrine	141	32.8
Open (outside)	85	19.8
Toilet distance		
Within 500 m	379	88.1
More than 500 m	51	11.9
Bathroom facility in household		
Attached	34	7.9
Detached	117	27.2
No bathroom	279	64.9
Facility for disposal of faeces of the children		
Diaper	9	2.1
Flush latrine	158	36.7
Garbage/dumping sites	263	61.2
Stagnant or sewage water near the house		
Yes	327	76
No	103	24
Wastewater network facility		
Yes	105	24.4
No	325	75.6
Solid waste piles near your house.		
Yes	116	73.02
No	314	26.98
Household waste management		
Throwing here and there	125	29.1
Have made our dustbin and put it in the side house	185	43
We deal with ourselves by burning them outside home	120	27.9

As per the study, 35.3% of households wash their hands with soap before eating and the same before cooking. Nearly 26% of household members bathe less than four days per week. The practise of hand washing with soap after using the toilet is found to be low (59.3%). Around 40% of households use footwear less often, 27% of households always use footwear and 33% of households never use footwear (Table 4).

**Table 4:** Frequency and percentage related to hygiene practices (n=430)

Hygiene factor	Frequency (n)	Percentage (%)
Hand washing frequency with soap		
Before food		
Yes	152	35.3
No	278	64.7
After food		
Yes	423	98.1
No	7	1.9
Before cooking		
Yes	152	35.3
No	278	64.7
After using toilet		
Yes	175	40.7
No	255	59.3
Bathing time of family members		
Every day	318	74
4 times or more per week	112	26
Habit of footwear		
Yes	116	27.0
No	142	33.0
Less often	172	40.0
Availability of appropriate material for menstruation		
Yes	388	90.2
No	42	9.8

In this study, 430 households with children aged between 6 months and 11 years participated. About one child in every five households (21.6%) has been suffering from diarrhoea in the last two weeks (Table 5).

**Table 5:** Frequency and percentage related to health (n=430)

Health factor	Frequency (n)	Percentage (%)
Occurrence of diarrhoea in past 2 weeks		
Yes	93	21.6
No	337	78.4

The study found an association in four variables with significance (P<0.05). The children of households near waste piles are more at risk of having diarrhoea than those households living far from the waste pile [AOR: 4.996; 95% CI: (2.173, 11.487)]. In the management of household wastes, household people who are throwing waste here and there are 3.276 times more at risk of having diarrhoea than those who are managing the wastes by themselves by burning them outside the home [AOR: 3.276; 95%CI:(1.463, 7.042)]. In the disposal of child faeces, it was found that the household children’s faeces disposed in the garbage or dumping site are 2.438 times more at risk of having diarrhoea than those who are flushing the faeces in the latrine [AOR: 2.438; 95%CI:(1.284, 4.631)]. Also, using footwear less often was found to be highly associated with an increase in the incidence of diarrhoea [AOR: 1.634; 95% CI (0.815, 3.276)] (Table 6).

**Table 6:** WASH factors associated with childhood diarrhoea (n=430)

WASH factors	Diarrhoea prevalence		Statistical tests	
	No n (%)	Yes n (%)	AOR (95%CI)	P-value
Principle source of drinking water				0.833
Supply water	224(66.5)	59(63.4)	0.941 (0.536, 1.651)	
Tube-well	113(33.5)	34(36.6)	Ref.	
Availability of drinking water from the principal source is sufficient throughout the year				0.654
No	55(16.3)	15(16.1)	1.186 (0.563, 2.498)	
Yes	282(83.7)	78 (83.9)	Ref.	
Drinking water taste				0.477
Good	97(28.8)	24(25.8)	803 (0.437, 1.472)	
Acceptable	240(71.2)	69(74.2)	Ref	
Water source for cooking				0.240
Domestic water	222(65.9)	64(68.8)	1.791 (0.909, 3.530)	
Drinking water	37(11)	11(11.8)	1.645 (0.631, 4.286)	
Both	78(23.1)	18(19.4)	Ref.	
Running water from the network per week				0.228
Not connected	59(17.5)	10(10.8)	0.446 (0.177, 1.120)	
4-7 days per week	153(45.4)	42(45.2)	0.791 (0.418, 1.497)	
2-3 days per week	125(37.1)	41(44)	Ref.	
Running water from the network per day				0.120
Not connected	59(17.5)	10(10.8)	0.568 (0.278, 1.159)	
Less than 4 hours per day	278(82.5)	83(89.2)	Ref.	
The amount of water you are receiving is enough to satisfy your needs				0.899
No	120(35.6)	35(37.6)	1.047 (0.517, 2.120)	
Yes	217(64.4)	58(62.4)	Ref.	
The distance to the principal source of drinking water				0.936
Within dwelling	53(15.7)	14(15.1)	1.017 (0.301, 3.438)	
Outside dwelling but within the premises	153(45.4)	41(44.1)	0.810 (0.281, 2.338)	
Outside premises: less than 0.2–0.5 km	107(31.8)	29(31.2)	827 (0.276, 2.476)	
More than 0.2–0.5 km	24(7.1)	9(9.7)	Ref	
Toilet facility in your household				0.800
Attached	37(11)	11(11.8)	1.219 (0.433, 3.429)	
Detached	226(67)	62(66.7)	1.271 (0.629, 2.569)	
No toilet	74(22)	20(21.5)	Ref.	
Toilet type				0.761
Pour latrine	159(47.2)	45(48.4)	0.906 (0.458, 1.790)	
Pit latrine	116(34.4)	25(26.9)	0.746 (0.332, 1.678)	
Open (outside)	62(18.4)	23(24.7)	Ref	
Toilet distance				0.207
Within 500 m	303(89.9)	76(81.7)	0.568 (0.235, 1.369)	
More than 500 m	34(10.1)	17(18.3)	Ref.	
Bathroom facility in household				0.965
Attached	26(7.7)	8(8.6)	1.016 (0.369, 2.796)	
Detached	90(26.7)	27(29)	1.088 (0.582, 2.036)	
No bathroom	221(65.6)	58(62.4)	Ref.	
Facility for disposal of faeces of the children				0.002
Garbage/dumping sites	115(34.1)	43(46.2)	2.438 (1.284, 4.631)	
Flush latrine	213(63.2)	50(53.8)	Ref.	
Stagnant or sewage water near house				0.215
No	77(22.8)	26(28)	1.487 (0.794, 2.783)	
Yes	260(77.2)	67(72)	Ref.	
Wastewater network facility				0.152
No	257(76.3)	68(73.1)	0.636 (0.342, 1.181)	
Yes	80(23.7)	25(26.9)	Ref.	
Solid waste piles near your house				0.001
No	107(31.8)	9(9.7)	Ref.	
Yes	230(68.2)	84(90.3)	4.996 (2.173, 11.487)	
Household waste management				0.004
Throwing here and there	91(27)	34 (36.6)	3.276 (1.463, 7.042)	
Have made our dustbin and put it inside house	143(42.4)	42(45.2)	1.061 (0.514, 2.190)	
We deal with ourselves by burning them outside home	103(30.6)	17(18.3)	Ref.	
Hand washing frequency with soap				
Before food				0.328
No	222(65.9)	56(60.2)	Ref.	
Yes	115(34.1)	37(39.8)	1.849 (0.120, 28.444)	
After food				0.685
No	6(1.8)	2(2.2)	Ref.	
Yes	331(98.2)	91(97.8)	1.166 (0.065, 20.984)	
Before cooking				0.807
No	219(65)	59(63.4)	Ref.	
Yes	118(35)	34(36.6)	1.209 (0.435, 3.361)	
After using toilet				0.405
No	196(58.2)	59(63.4)	Ref.	
Yes	141(41.8)	34(36.6)	1.502 (0.566, 3.986	
Bathing time of family members				0.455
Every day	250(74.2)	68(73.1)	0.797 (0.439, 1.446)	
Four times or more per week	87(25.8)	25(26.9)	Ref.	
Habit of footwear				0.001
No	107(31.8)	9(9.7)	0.225 (0.089, 0.569)	
Yes	130(38.6)	42(45.2)	Ref	
Less often	100(29.7)	42(45.2)	1.634 (0.815, 3.276)	
Availability of appropriate material for menstruation				0.513
No	306(90.8)	82(88.2)	0.743 (0.306, 1.808)	
Yes	31(9.2)	11(11.8)	Ref.	

## Discussion

Findings from the present study illustrated that there was a strong association between WASH practises and childhood diarrhoea. Children who were living in unhygienic conditions or whose parents were following unhygienic practises were more at risk of having childhood diarrhoea. In the year 2015, Lakshminarayanan and Jayalakshmy suggested that poor socioeconomic status, maternal educational qualification, breastfeeding, malnutrition, sanitation, and hygiene practises of the mother were associated with an increase in the incidence of diarrheal diseases in younger children [25]. Hand cleaning while making meals and caring for their children, as well as indiscriminate dumping of under five children's faeces by mothers, must be addressed [26]. A low level of mother literacy was found to have a positive connection with an increase in the frequency of childhood diarrhoea [27].

Although 45% of the households had a water supply, it was still not a continuous supply, while 16% of the households did not have a water network or supply. So it is obvious that hygiene is difficult to maintain. Wolf et al. found that the risk of diarrhoeal morbidity can be prevented by safe storage of water, piped water to premises of higher quality, and continuous availability [28].

Our study results reported that the majority of kids' faeces were dumped in garbage or dumping sites. This might be due to the fact that the majority of caregivers or mothers are illiterate, and toilets are not readily available. Around 90.2% of families had no suitable material to be used by girls during menstruation. This finding is consistent with a study that found 80% of Indian women do not have access to basic menstrual protection [29]. It was found that children of households near waste piles are 4.996 times more at risk of having diarrhoea than those whose households are not near waste piles. Children from families that threw solid trash around (here and there) and those who constructed their own dustbins and placed them inside the house were 3.210 and 1.061 times more likely to get diarrhoea, respectively, than those who dealt with solid waste by burning it. It reflects that the members of those households are clearly unaware of the fact that the waste they are throwing into their environment can harm their children`s health by causing illnesses like diarrhoea. As the study conducted in the year 2019 showed that dumping kid’s faeces at garbage sites or random dumping locations was triggering diarrhoea in under five (U5) children, it means that environmental hygiene factor is a key indicator to focus on for diarrhoea prevention in under five children [30]. A study done in Ethiopia in the year 2020 among children suggested the same findings: that environmental sanitation is one of the risk factors to be addressed in order to keep children's nutritional status healthy [11]. In another study on diarrhoea and waste disposal in Salvador, Brazil, in the year 2005, it was discovered that the estimated diarrhoea prevalence was 21.2%. Environmental garbage exposure was shown to be the most significant [31]. Hence, it is necessary to monitor the WASH-related activities that are taking place in rural settings and also to develop awareness about WASH among the rural people. The frontline workers can be trained and used as WASH activists in order to bring complete hygiene among rural households to prevent their children from diarrhoea. Household members who wore shoes less frequently were 1.634 times more likely to get diarrhoea than those who wore shoes when going outside. A meta-analysis conducted by Tomczyk and Deribe in 2014 to find the association between the use of footwear and neglected tropical diseases found that footwear use was significantly associated with lower odds of infection of Buruli ulcer, tungiasis, hookworm infection, any soil-transmitted helminth (STH) infections, strongyloidiasis, and leptospirosis [32].

The type of latrine owned by that particular household had a significant influence on the occurrence of diarrhoea. People whose households are not connected to the water pipe system may find it difficult to build and use a flush toilet. As a result, pour toilets and pit latrines are more commonly used in the study area. The findings from this study are similar to the findings from the studies conducted in India and Tanzania, which revealed more use of pour toilets and pit toilets than flush toilets due to the inaccessibility of the water system [33-34]. Therefore, the occurrence of diarrhoea is likely to be higher in households who use pour latrines and pit latrines than in others. Hence, it is essential to address each component of WASH and educate the people on the health benefits of having good sanitation/improved latrines.

The study findings showed that the prevalence of diarrhoea is likely to be more in households who had stagnant or sewage near their house. It was also revealed that the diarrhoea cases are more common in households who made their own dustbins and who dump their household waste here and there. These findings are consistent with a study conducted in 2017, Vellore, Chennai revealed that the prevalence of the infection is lower in households with proper sludge management, compared with children in households with toilets with poor sludge management. They further mentioned that children are more likely to have regular contact with the floors and grounds both inside and outside the home, all of which may expose them to dirt and infection from the outside environment, either directly or indirectly [35]. However, while the precise exposure pathway is unknown, there are several ways in which infected pathogens emitted from sludge areas may affect young children. To reduce diarrheal illness and improve health, toilet coverage must accompany safe sewage management with good sanitation and hygiene practices.

Study limitations

Being a cross-sectional study, the cause-effect relationship between diarrhoea and WASH practises could not have been established. Generalization of the study is limited as it is carried out in a specific region. In addition, diarrhoea-associated infections may have a seasonal effect, which may cause reporting bias.

## Conclusions

The present study illustrated the reason for diarrhoea prevalence in the study area by aggregating the data. Unsafe drinking water, poor sanitation and hygiene practises are the risk factors for developing childhood diarrhoea. Therefore, it is important for region-specific resource allocation to prevent childhood diarrhoea. Also, priority should be given to maintaining periodic monitoring of households by local authorities and conducting counselling of mothers for improved household WASH practices. Counselling on preventive practises like proper household waste management and using footwear needs to be prioritized. In addition, proper household sanitation facilities, required nutritional intake for both child and mother, safe disposal practices of faeces, vaccination for rotavirus and careful diarrhoeal case management are some of the effective interventions to be implemented. Along with these, more research studies regarding cause and effect relationships between WASH practises and the prevalence of diarrhoea, aetiology, and risk factors should be carried out. Moreover, evidence-based policies need to be formulated and rolled out in the country to sustain diarrhoeal prevention programs.
